# Safety and tolerability of intravenous regadenoson in healthy subjects: A randomized, repeat-dose, placebo-controlled study

**DOI:** 10.1007/s12350-015-0327-9

**Published:** 2015-11-25

**Authors:** Robert Townsend, Amit Desai, Diane Rammelsberg, Donna Kowalski, Neal Simmons, Therese M. Kitt

**Affiliations:** 1 Astellas Pharma Global Development, Inc., 1 Astellas Way, Northbrook, IL 60062 USA; 2 Randstad Pharma, Deerfield, IL USA; 3 Astellas Research Institute of America, Bioanalysis-US, Skokie, IL USA

**Keywords:** Myocardial perfusion imaging, regadenoson, repeated doses, safety, tolerability

## Abstract

**Background:**

Regadenoson is a selective A_2A_ adenosine receptor agonist indicated for radionuclide myocardial perfusion imaging in patients unable to undergo adequate exercise stress. However, the safety, tolerability, and plasma concentrations associated with repeated doses have not previously been assessed.

**Method and Results:**

Healthy males and females were randomized to receive intravenous regadenoson [100 μg (3 doses), 200 μg (3 doses), or 400 μg (2 doses)], or placebo (2 or 3 doses; 0.9% sodium chloride); all doses 10 minutes apart. The primary endpoint was vital sign measurements (blood pressure and heart rate). Secondary endpoints included 12-lead electrocardiogram measurements, clinical laboratory evaluations (hematology, chemistry, and urinalysis), and adverse events. Thirty-six subjects were randomized and completed the study. Plasma concentrations of regadenoson increased in a dose-related manner and with successive doses. No consistent effect was observed for systolic blood pressure, although diastolic blood pressure was slightly lower than placebo for all regadenoson groups. Transient, dose-dependent increases in heart rate were observed in all regadenoson groups. There were no serious adverse events; 27 adverse events occurred in 14 regadenoson-treated subjects vs two events in two placebo-treated subjects.

**Conclusion:**

Repeated doses of regadenoson appeared to be safe and well tolerated in healthy subjects.

## Introduction

Patients who are unable to perform adequate exercise stress for myocardial perfusion imaging (MPI) are generally administered a pharmacologic stress agent such as adenosine, dipyridamole, or regadenoson.^1^ Adenosine and regadenoson exert dilatory effects on the coronary vasculature via activation of the A_2A_ adenosine receptor, but adenosine can also cause unwanted systemic vascular and hemodynamic effects primarily via activation of other adenosine receptor subtypes. Regadenoson is a selective A_2A_ adenosine receptor agonist indicated for radionuclide MPI in patients unable to undergo adequate exercise stress.^2^ In phase 3 clinical studies, single doses of regadenoson were found to be equally efficacious as adenosine.^3^,^4^ The safety and tolerability of regadenoson have also been confirmed in various special populations of patients, including those with kidney, liver and airway diseases.^5^-^9^

During intravenous (IV) administration, regadenoson may infiltrate into subcutaneous tissues in the arm, thus requiring cessation of delivery and the possibility of repeating the procedure. The safety and tolerability of repeat consecutive doses of regadenoson, as well as the associated plasma concentrations, have not been systematically assessed. In the present study, we evaluated these parameters in healthy supine subjects after IV bolus doses of 100, 200, and 400 μg repeated up to three times at 10-minute intervals.

The study will provide information on the safety and tolerability of re-dosing regadenoson.

## Methods

This was a randomized, double-blind, dose escalation, parallel-group, placebo-controlled, repeat dose-tolerance clinical study of regadenoson in healthy, nonsmoking subjects performed in the United States between May and August 2013 (ClinicalTrials.gov identifier, NCT01918995). The study was approved by an institutional review board and was conducted in accordance with Good Clinical Practice and International Conference on Harmonization guidelines.^10^

### Subjects

Eligible subjects were healthy men and women, aged 18-60 years with a body weight of ≥45 kg and body mass index between 18 and 32 kg/m^2^, both inclusive; Fridericia-corrected QT interval of ≤430 msec (males) or ≤450 msec (females); and normal aspartate aminotransferase, alanine aminotransferase, and total bilirubin levels. Female subjects of childbearing potential were required to use highly effective birth control from Screening through 28 days after the end of the study, and male subjects were required to use highly effective contraception from Screening through 90 days after final study drug administration. Exclusion criteria included a history of unexplained syncope, cardiac arrest, unexplained cardiac arrhythmia or torsade de pointes, structural heart disease, or family history of either short or long QT syndrome; a positive result for hepatitis B surface antigen, hepatitis C antibodies at screening or known human immunodeficiency virus infection; known or suspected allergy to regadenoson or any of the components of the trial products, or a history of multiple and/or severe allergies to drugs or foods, or a history of severe anaphylactic reactions; a history of smoking within 6 months prior to first dose of regadenoson; treatment with any prescribed or nonprescribed drugs in the 2 weeks prior to day 1 (except occasional use of acetaminophen, ≤2 g/day); participation in any interventional clinical study or treatment with any investigational drugs within 30 days or five half-lives, whichever was longer, prior to screening; participation in a prior study with regadenoson; or history of consuming more than 14 units of alcoholic beverages per week within 6 months prior to screening or history of alcoholism or drug/chemical/substance abuse within past 2 years prior to screening or a positive test at screening or 1 day prior to study start for alcohol or drugs of abuse.

### Study Design and Assessments

Subjects were randomized to receive IV regadenoson [100 μg (3 doses), 200 μg (3 doses), or 400 μg (2 doses)] or placebo (2 or 3 doses; 0.9% sodium chloride) at a ratio of 3:1 (regadenoson to placebo) for each group. Subjects were required to abstain from eating and drinking (except water) for 8 hours prior to drug administration. Subjects received each dose (over approximately 10 s) in a supine position administered 10 minutes apart followed by a 5 mL 0.9% saline flush. The first cohort (9 regadenoson, 3 placebo) started with the lowest dose of regadenoson, and after a favorable review of safety, the regadenoson dose was escalated stepwise in subsequent cohorts.

Blood samples for pharmacokinetic analyses were obtained prior to study drug administration and at 3, 9, 13, 19, 23, and 29 minutes, and at 1, 2, 4, 8, and 12 hours after the initial administration. Serial blood pressure and heart rate measurements for pharmacodynamic analyses were obtained prior to dosing, at 1, 3, 5, 7, 9, 11, 13, 17, 19, 21, 23, 25, 27, and 29 minutes during dosing, and at 0.75, 1, 1.5, 2, 4, 8, 12, and 24 hours postdose. Using the maximum and/or minimum values for all post-baseline assessments, hemodynamic effects were defined using the following criteria: heart rate of ≥100 bpm or an increase of >40 bpm; systolic blood pressure of <90 mm Hg or ≥200 mm Hg, or decreased by >35 mm Hg, or increased by ≥50 mm Hg, or ≥180 mm Hg at baseline and an increase of ≥20 mm Hg; diastolic blood pressure of <50 mm Hg or ≥115 mm Hg, or decreased by >25 mm Hg, or increased by ≥30 mm Hg.

The primary endpoint of the study was safety as evaluated by vital signs (blood pressure and heart rate). Secondary endpoints included 12-lead electrocardiogram (ECG) measurements, clinical laboratory evaluations (hematology, chemistry, and urinalysis), and adverse events (AEs). Plasma concentrations of regadenoson were included as an exploratory endpoint.

### Bioanalysis

Frozen plasma samples, containing sodium heparin as the anticoagulant, were shipped to Worldwide Clinical Trials (Austin, TX, USA) for analysis using a validated LC-MS/MS method. Regadenoson was measured over the calibration range 0.1-40 ng/mL using a 0.2 mL plasma sample volume and a stable isotope-labeled internal standard (d_3_-regadenoson). Sample purification used Strata-X (Phenomenex, Torrance, CA, USA) 10 mg solid phase extraction 96-well plates followed by chromatographic separation on a Luna C18(2) 3 µm 2 × 20 mm HPLC column (Phenomenex, Torrance, CA). The elution profile was a gradient of acetonitrile and water-containing formic acid. A Sciex API5000 (Applied Biosystems, Foster City, CA, USA) mass spectrometer was used to monitor the mass transitions (m/z) 391 → 259 (regadenoson) and 394 → 262 (internal standard) in positive TurboIonSpray™ mode. The mean bias and precision of each quality control level was within −6.3% to −1.4%, and 1.1% to 3.4%, respectively, over the four analytic runs of this study.

### Statistical Analysis

A sample size of 36 subjects (regadenoson, 9 per treatment group; placebo, 3 per treatment group) was selected. Three analysis sets were used; the safety analysis set (all subjects who were randomized and received ≥1 dose of study drug), the pharmacokinetic analysis set (all subjects in the safety analysis set who had ≥1 quantifiable concentration, and for whom no major clinical study protocol deviation that was thought to have interfered with the distribution, metabolism, and excretion of the compound measured had been reported), and the pharmacodynamic analysis set (all subjects in the safety analysis set who provided ≥1 blood pressure or heart rate measurement postdose on day 1; subjects with missed doses or protocol violations were assessed on a subject-by-subject basis for inclusion). Pharmacokinetic and pharmacodynamic data were assessed using descriptive statistics (mean ± standard deviation).

## Results

### Subject Disposition and Demographics

**Figure 1:**
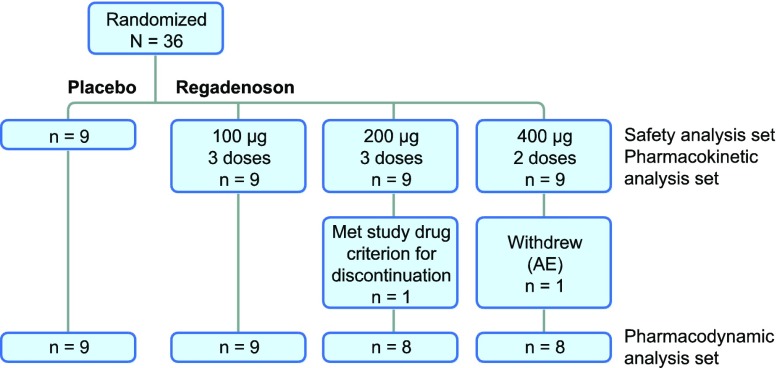
Subject disposition flow diagram

In total, 36 subjects were randomized and all completed the study (Figure 1). Twenty (55.6%) subjects were male, and the mean age of all subjects was 37.7 years (Table 1). A greater percentage of black or African American subjects were randomized to the placebo group, whereas the regadenoson groups included similar percentages of black or African American subjects and white subjects. One subject each in the 200- and 400-μg regadenoson groups discontinued treatment after the first planned dose (see Adverse events, below). These subjects presented quantifiable plasma concentrations up to 4 and 8 hours, respectively, but because they did not receive the subsequent planned doses, plasma concentrations measured more than 9 minutes after the initial dose (i.e., after the second planned dose) were excluded from the summary statistics, and data from both subjects were excluded from the pharmacodynamics analysis set.

**Table 1:** Demographics and baseline characteristics

Parameter	Placebo *n* = 9	Regadenoson			Total *n* = 36
		100 μg *n* = 9	200 μg *n* = 9	400 μg *n* = 9	
Sex [*n* (%)]					
Male	6 (66.7)	4 (44.4)	4 (44.4)	6 (66.7)	20 (55.6)
Female	3 (33.3)	5 (55.6)	5 (55.6)	3 (33.3)	16 (44.4)
Race [*n* (%)]					
White	1 (11.1)	8 (88.9)	2 (22.2)	4 (44.4)	15 (41.7)
Black or African American	7 (77.8)	1 (11.1)	7 (77.8)	5 (55.6)	20 (55.6)
Asian	1 (11.1)	0	0	0	1 (2.8)
Ethnicity [*n* (%)]					
Hispanic or Latino	1 (11.1)	3 (33.3)	2 (22.2)	2 (22.2)	8 (22.2)
Non-Hispanic or Latino	8 (88.9)	6 (66.7)	7 (77.8)	7 (77.8)	28 (77.8)
Age (years)					
Mean (SD)	36.1 (8.0)	38.3 (11.2)	43.2 (11.3)	32.7 (10.6)	37.7 (10.7)
Range	21–51	25–56	26–60	22–58	21–60
Height (cm)					
Mean (SD)	172.4 (8.6)	171.2 (10.9)	170.6 (11.2)	170.1 (12.7)	171.1 (10.5)
Range	155–186	158–186	157–183	156–195	155–195
Weight (kg)					
Mean (SD)	77.9 (15.8)	69.8 (10.3)	79.5 (13.5)	73.1 (14.8)	75.1 (13.7)
Range	60.8–106.3	52.1–86.2	58.1–95.1	47.7–87.3	47.7–106.3
BMI (kg/m^2^)					
Mean (SD)	26.0 (3.5)	23.7 (1.9)	27.2 (3.1)	25.1 (3.6)	25.5 (3.2)
Range	21.3–31.9	20.9–26.3	22.2–31.6	19.6–30.8	19.6–31.9

*BMI* body mass index, *SD* standard deviation

### Pharmacodynamic Results

**Figure 2:**
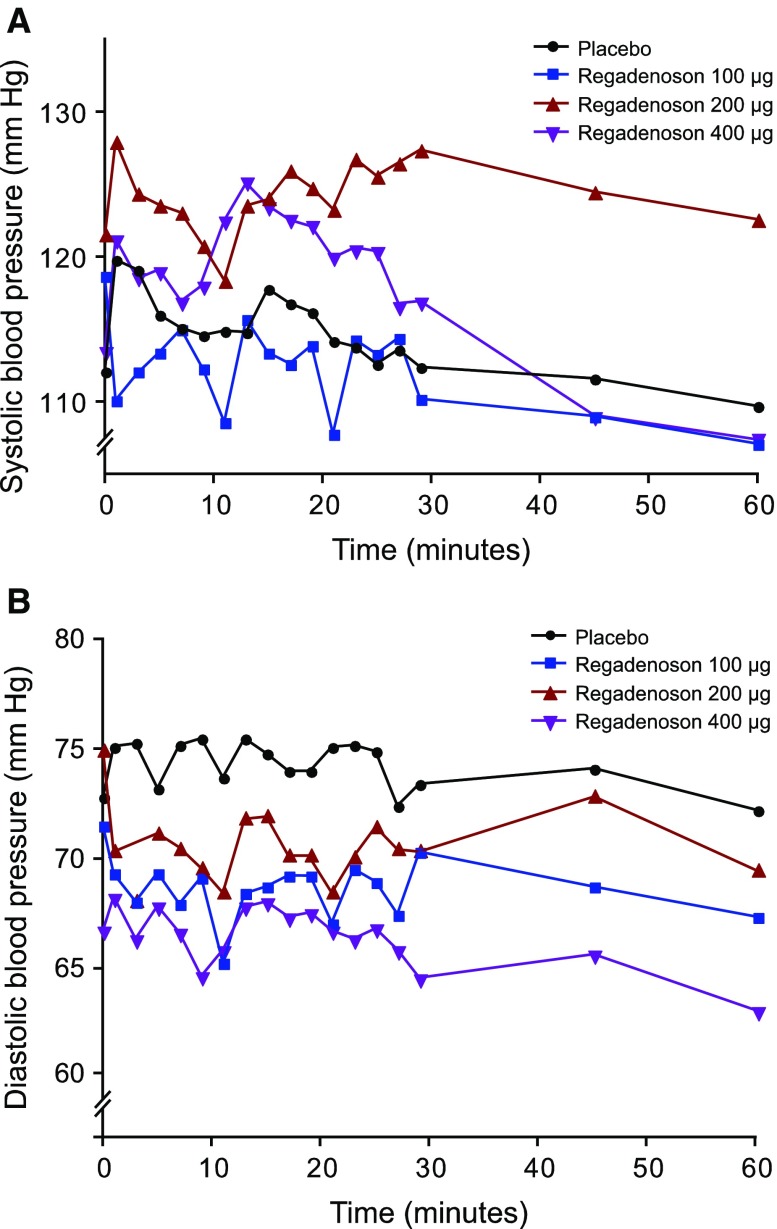
Mean observed blood pressure up to 1 hour postdose. (**A**) Systolic blood pressure; (**B**) diastolic blood pressure

Overall, there was no consistent pattern of effects on systolic blood pressure associated with repeat-dose administration of regadenoson (Figure 2A, Appendix Table 5). Although the mean systolic blood pressure for the 200-μg group remained slightly elevated compared with placebo, this appeared to reflect a higher baseline value for this group (121.6 mm Hg vs 112.1 mm Hg, respectively), and no consistent differences with placebo were observed for the 100-μg and 400-μg groups. Repeat dosing with regadenoson was associated with small reductions in diastolic blood pressure (in general, mean change of <10 mm Hg) (Figure 2B, Appendix Table 5).

**Figure 3:**
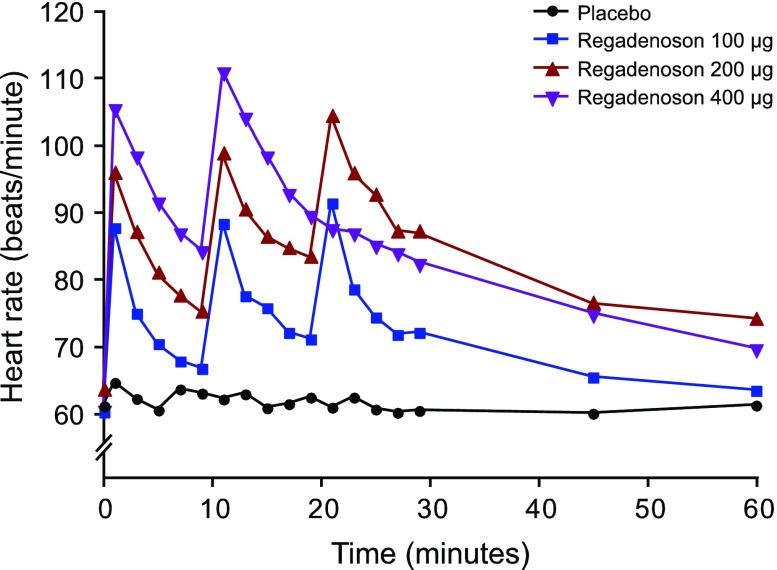
Mean observed heart rate up to 1 hour postdose

Transient dose-dependent increases in heart rate occurred following administration of each dose of regadenoson (Figure 3, Appendix Table 6). At 1 minute postdose (i.e., the first measured time), the mean heart rate was increased in the 100-μg group (increase of 28-31 bpm), 200-μg group (increase of 32-42 bpm), and the 400-μg group (increase of 45-51 bpm).

Categorical hemodynamic effects for blood pressure and heart rate were predefined (see “Methods” section). There was no obvious dose-dependence for effects on systolic or diastolic blood pressure in the categorical assessment, although effects on heart rate were more frequently observed with higher doses (Table 2). No subject in the placebo group met any of the predefined criteria for hemodynamic effects.

**Table 2:** Blood pressure and heart rates for subjects with hemodynamic effects

Parameter/ criteria [*n* (%)]	Placebo *n* = 9	Regadenoson		
		100 μg *n* = 9	200 μg *n* = 8	400 μg *n* = 8
Systolic blood pressure				
<90 mm Hg	0	3 (33.3)	0	0
≥200 mm Hg	0	0	0	0
Decrease >35 mm Hg	0	2 (22.2)	1 (12.5)	0
Increase ≥115 mm Hg	0	0	0	0
≥80 and increase ≥20 mm Hg	0	0	0	0
Diastolic blood pressure				
<50 mm Hg	0	1 (11.1)	0	1 (12.5)
≥115 mm Hg	0	0	0	0
Decrease >25 mm Hg	0	1 (11.1)	1 (12.5)	0
Increase ≥30 mm Hg	0	0	0	0
Heart rate				
≥100 bpm	0	3 (33.3)	7 (87.5)	6 (75.0)
Increase >40 bpm	0	2 (22.2)	4 (50.0)	7 (87.5)

All data from the pharmacodynamic population

### Regadenoson Concentration-Time Profiles

**Figure 4:**
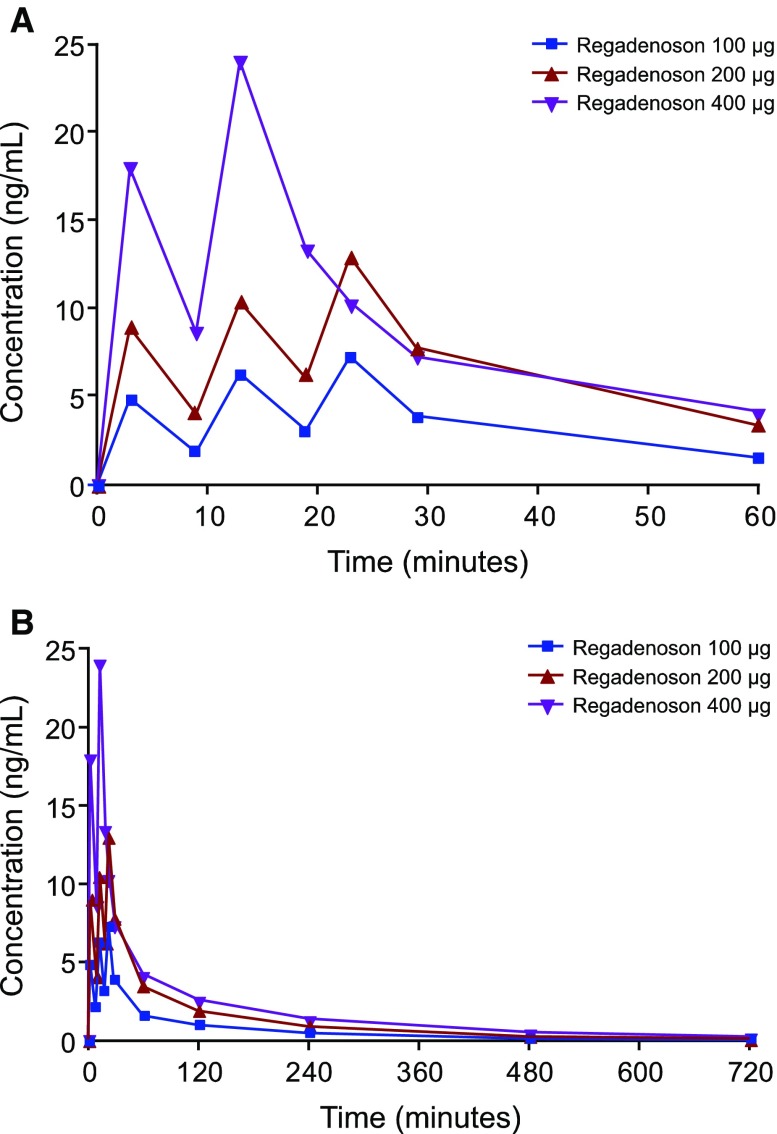
Mean plasma regadenoson concentration-time profiles over 60 min (**A**) and 12 h (**B**)

The mean plasma concentration-time profiles of regadenoson obtained are shown in Figure 4A, B (Appendix Table 7). In each of the 100-, 200-, and 400-μg groups, the mean concentrations reached a rapid peak after administration of each dose followed by a relatively slower decline. The mean peak concentrations increased in a dose-related manner and increased slightly with each successive dose. After 12 hours, plasma levels fell below the level of quantification for all subjects in the 100-μg group, for four subjects in the 200-μg group, and for three subjects in the 400-μg group.

### Adverse Events

A total of 27 AEs were reported for 14 subjects receiving regadenoson (51.9%), whereas two AEs were reported for two subjects receiving placebo (22.2%) (Table 3). Fourteen AEs were reported for eight (88.9%) subjects in the 400-μg group, 13 AEs were reported for six (66.7%) subjects in the 200-μg group, and no AEs were reported in the 100-μg group. Headache, nausea, dizziness, sweating, and feeling hot were the most commonly reported AEs occurring in ≥5% of the total subjects receiving regadenoson. All AEs were considered by investigators to be mild or moderate in severity.

**Table 3:** Reported adverse events*

Number of subjects with adverse events [*n* (%)]	Placebo *n* = 9	Regadenoson			
		100 μg *n* = 9	200 μg *n* = 9	400 μg *n* = 9	Total *n* = 27
Any adverse event	2 (22.2)	0	6 (66.7)	8 (88.9)	14 (51.9)
Adverse events leading to study drug discontinuation	0	0	0	1 (11.1)	1 (3.7)
Headache	1 (11.1)	0	5 (55.6)	4 (44.4)	9 (33.3)
Nausea	0	0	1 (11.1)	2 (22.2)	3 (11.1)
Dizziness	0	0	1 (11.1)	1 (11.1)	2 (7.4)
Sweating	0	0	1 (11.1)	1 (11.1)	2 (7.4)
Feeling hot	0	0	0	2 (22.2)	2 (7.4)
Palpitations	0	0	0	1 (11.1)	1 (3.7)
Stomach discomfort	0	0	0	1 (11.1)	1 (3.7)
Anxiety	0	0	0	1 (11.1)	1 (3.7)
Dyspnea	0	0	0	1 (11.1)	1 (3.7)
Dysphagia	0	0	1 (11.1)	0	1 (3.7)
Muscular weakness	0	0	1 (11.1)	0	1 (3.7)
Paresthesia	0	0	1 (11.1)	0	1 (3.7)
Somnolence	0	0	1 (11.1)	0	1 (3.7)
Subcutaneous nodule	1 (11.1)	0	0	0	0

* Any adverse event observed after administration of the first dose of study drug or matching placebo until 24 hours after administration of the last dose of study drug or matching placebo

There were no serious AEs or deaths, and no clinically significant findings were observed in ECGs or clinical laboratory assessments. Two subjects discontinued treatment after the first study dose of regadenoson. One subject in the 400-μg group withdrew consent to receive further treatment due to anxiety and palpitations. One subject in the 200-μg group met the protocol-defined criteria for discontinuation of decreased systolic blood pressure (two consecutive measurements of <100 mm Hg). When the incidence of AEs was assessed following each sequential dose, there was no obvious pattern of their occurrence with respect to each dose (Table 4).

**Table 4:** Reported adverse events following each dose

Event following	Placebo *n* = 9	Regadenoson		
		100 μg *n* = 9	200 μg *n* = 9	400 μg *n* = 9
Dose 1 [*n* (%)], E	0	0	4 (44.4), 7	5 (55.6), 9
AE			2 headache	2 feeling hot
			1 paraesthesia	1 dyspnoea
			1 dizziness	1 hyperhidrosis
			1 dysphagia	1 anxiety
			1 nausea	1 palpitations
			1 hyperhidrosis	1 dizziness
				1 headache
				1 nausea
Dose 2 [*n* (%)], E	1 (11.1), 1	0	4 (44.4), 4	4 (44.4), 5
AE	1 headache		2 headache	3 headache
			1 somnolence	1 stomach discomfort
			1 muscular weakness	1 nausea
Dose 3 [*n* (%)], E	1 (11.1), 1	0	1 (11.1), 2	NA
AE	1 subcutaneous nodule		1 headache	

*N* number of subjects, *E* number of events, *NA* not applicable

## Discussion

This study demonstrated that, following repeated IV administration of regadenoson at doses of 100, 200, or 400 μg, the heart rate was transiently increased, whereas the diastolic blood pressure was slightly lower than with placebo. Although the dose-related effect on heart rate paralleled the measurable plasma concentration, the effect on diastolic blood pressure was not clearly dose dependent, and no consistent effect was evident on systolic blood pressure in these subjects. Repeated doses were well tolerated in these subjects, as AEs were not experienced by subjects in the 100-μg group and no unexpected AEs were reported in the 200- and 400-μg groups.

The increases in heart rate in this study were greater than those observed in the phase 3 clinical studies, perhaps owing to factors such as the younger age and lack of cardiovascular disease in the healthy subjects included in this study. Differences in the responses of heart rate and blood pressure following administration of regadenoson may provide further support for the idea that A_2A_ receptor agonists may exert effects on the sympathetic nervous system that are separable from effects on vascular baroreceptors. For example, intracerebral administration of a selective A_2A_ receptor agonist in rats was shown to increase heart rate without any accompanying effect on blood pressure.^11^ In a separate study, the increased heart rate in rats treated with regadenoson was shown to be inhibited by metoprolol (a *β* blocker), whereas regadenoson-mediated effects on blood pressure were unaffected.^12^ Since some AEs such as chest pain and dyspnea associated with adenosine have also been observed with regadenoson (albeit at a lower incidence), it had been proposed that these effects might be due at least in part to sympathetic stimulation rather than activation of different adenosine receptor subtypes.^4^ An indirect effect via sympathetic nerves may also help to explain the lack of clear dose-relatedness in blood pressure responses, because sympathetic activity would also be subject to regulation via multiple other receptors.

The study is limited by the relatively small sample size in each treatment group, which limits the conclusions that can be drawn. For example, it is possible that dose-related effects on blood pressure may have been more evident with larger study groups. Moreover, the results in healthy subjects cannot necessarily be extrapolated to patients for whom MPI would be indicated. Nevertheless, the efficacy, safety, and tolerability of single doses of regadenoson have been demonstrated to be independent of various baseline factors in patients who require MPI.^3^ Based on the current data, there is no reason to believe that these factors would necessarily differ with repeated doses.

Importantly, the observed plasma concentration and pharmacodynamic effects of repeated doses of regadenoson in this study were predictable based on these parameters in single-dose studies.^3^,^4^,^13^ Given the consistency of safety and tolerability also between single and repeated doses, this study may further support the use of regadenoson for MPI in subjects unable to undergo exercise stress.

### New Knowledge Gained

In healthy subjects, doses of regadenoson repeated up to three times (100 or 200 μg) or two times (400 μg) at 10-minute intervals produce dose-related increases in regadenoson plasma concentrations and heart rate. Systolic blood pressure fell by 9-11 mmHg following each dose of regadenoson 100 µg and increased by 8-9 mmHg following each dose of regadenoson 400 µg. These repeated doses appeared to be safe and consistent with the known adverse event profile of regadenoson.

## Appendix

See Tables 5, 6, and 7.

**Table 5:** Mean (SD) observed systolic blood pressure (SBP) and diastolic blood pressure (DBP) up to 1 hour postdose in the pharmacodynamic analysis set

Time (min)	Placebo Mean mm Hg (SD)		Regadenoson 100 µg Mean mm Hg (SD)		Regadenoson 200 µg Mean mm Hg (SD)		Regadenoson 400 µg Mean mm Hg (SD)	
	SBP	DBP	SBP	DBP	SBP	DBP	SBP	DBP
0	112.1 (9.2)	72.7 (7.1)	118.7 (9.1)	71.4 (9.0)	121.6 (10.2)	74.9 (13.5)	113.4 (7.1)	66.5 (8.4)
1	119.8 (11.1)	75.0 (5.9)	110.1 (18.5)	69.2 (9.5)	128.0 (9.4)	70.3 (7.5)	121.1 (11.9)	68.0 (8.5)
3	119.1 (9.8)	75.2 (6.9)	112.1 (9.9)	67.9 (7.4)	124.4 (7.0)	68.0 (8.6)	118.5 (9.5)	66.1 (6.7)
5	116.0 (8.7)	73.1 (6.3)	113.4 (9.7)	69.2 (7.6)	123.6 (8.0)	71.1 (7.2)	118.9 (10.4)	67.6 (8.4)
7	115.1 (8.8)	75.1 (6.1)	115.0 (11.9)	67.8 (8.0)	123.1 (4.9)	70.4 (7.7)	116.8 (9.8)	66.4 (7.9)
9	114.6 (7.3)	75.4 (7.5)	112.3 (11.8)	69.0 (5.5)	120.8 (5.2)	69.5 (7.9)	117.9 (8.9)	64.4 (9.1)
11	114.9 (10.5)	73.6 (6.3)	108.6 (17.1)	65.1 (11.4)	118.4 (12.1)	68.4 (6.3)	122.4 (16.9)	65.6 (9.9)
13	114.8 (8.0)	75.4 (5.6)	115.7 (11.4)	68.3 (6.5)	123.6 (8.5)	71.8 (9.8)	125.1 (13.0)	67.6 (10.1)
15	117.8 (5.9)	74.7 (7.0)	113.4 (11.3)	68.6 (6.1)	124.1 (7.6)	71.9 (8.3)	123.4 (10.4)	67.8 (10.7)
17	116.8 (7.6)	73.9 (6.5)	112.6 (7.3)	69.1 (5.9)	126.0 (8.5)	70.1 (7.9)	122.5 (11.0)	67.1 (9.3)
19	116.2 (8.1)	73.9 (5.1)	113.9 (10.0)	69.1 (6.0)	124.8 (8.3)	70.1 (8.5)	122.1 (10.5)	67.3 (8.1)
21	114.2 (6.1)	75.0 (5.5)	107.8 (16.2)	66.9 (9.8)	123.3 (6.1)	68.4 (7.2)	119.9 (11.7)	66.5 (9.0)
23	113.8 (6.0)	75.1 (4.0)	114.3 (11.2)	69.4 (7.4)	126.8 (6.2)	70.0 (9.7)	120.4 (8.2)	66.1 (7.0)
25	112.6 (7.4)	74.8 (4.7)	113.3 (7.9)	68.8 (8.2)	125.6 (6.0)	71.4 (9.3)	120.3 (11.1)	66.6 (8.6)
27	113.6 (7.7)	72.3 (4.7)	114.4 (8.8)	67.3 (6.6)	126.5 (7.0)	70.4 (8.5)	116.5 (8.6)	65.6 (8.6)
29	112.4 (6.8)	73.3 (5.2)	110.2 (6.9)	70.2 (8.9)	127.4 (8.3)	70.3 (8.1)	116.8 (11.0)	64.3 (7.3)
45	111.6 (7.6)	74.0 (6.6)	109.0 (7.3)	68.6 (7.4)	124.5 (7.6)	72.8 (11.0)	108.9 (8.7)	65.4 (4.9)
60	109.7 (9.0)	72.1 (7.0)	107.1 (5.7)	67.2 (5.9)	122.6 (7.4)	69.4 (7.8)	107.4 (8.7)	62.8 (6.8)

**Table 6:** Mean (SD) observed heart rate up to 1 hour postdose in the pharmacodynamic analysis set

Time (min)	Placebo Mean beats/minute (SD)	Regadenoson 100 µg Mean beats/minute (SD)	Regadenoson 200 µg Mean beats/minute (SD)	Regadenoson 400 µg Mean beats/minute (SD)
0	61.3 (12.1)	60.4 (12.3)	63.9 (10.1)	60.4 (14.7)
1	64.8 (11.2)	88.0 (8.1)	96.4 (16.7)	105.6 (17.2)
3	62.4 (9.6)	75.2 (14.0)	87.5 (18.3)	98.5 (16.7)
5	60.7 (9.0)	70.6 (12.2)	81.4 (13.5)	91.6 (14.1)
7	63.8 (10.9)	68.0 (13.3)	78.0 (11.5)	87.0 (16.3)
9	63.2 (10.2)	66.9 (13.1)	75.6 (12.0)	84.3 (13.5)
11	62.3 (10.3)	88.6 (10.7)	99.3 (13.3)	111.1 (19.1)
13	63.1 (8.0)	77.8 (15.3)	90.9 (13.5)	104.3 (17.3)
15	61.0 (9.4)	76.0 (16.1)	86.8 (11.5)	98.5 (19.8)
17	61.6 (9.8)	72.3 (14.0)	85.1 (12.8)	92.9 (17.0)
19	62.6 (8.1)	71.3 (14.4)	83.8 (11.3)	89.6 (18.0)
21	61.1 (10.0)	91.7 (9.5)	104.9 (10.7)	87.6 (17.3)
23	62.6 (11.1)	78.8 (13.7)	96.3 (13.0)	87.0 (15.5)
25	60.8 (9.9)	74.6 (13.0)	93.1 (10.1)	85.1 (18.6)
27	60.3 (9.4)	72.0 (11.9)	87.6 (11.2)	84.0 (14.3)
29	60.6 (10.4)	72.3 (14.3)	87.6 (8.4)	82.4 (15.7)
45	60.2 (10.5)	65.6 (11.3)	76.9 (11.0)	74.8 (13.6)
60	61.4 (12.2)	63.6 (11.1)	74.5 (11.3)	69.6 (12.3)

**Table 7:** Mean (SD) plasma regadenoson concentration-time profiles over 12 hours in the pharmacokinetic analysis set

Time (minutes)	Regadenoson 100 µg Mean ng/mL (SD)	Regadenoson 200 µg Mean ng/mL (SD)	Regadenoson 400 µg Mean ng/mL (SD)
0	0 (0)	0 (0)	0 (0)
3	4.909 (1.177)	9.048 (2.554)	17.948 (6.205)
9	2.001 (0.275)	4.103 (0.627)	8.567 (1.416)
13	6.334 (1.249)	10.486 (2.663)	23.991 (5.493)
19	3.132 (0.419)	6.235 (1.033)	13.370 (2.314)
23	7.346 (1.199)	13.005 (3.628)	10.233 (2.192)
29	3.928 (0.624)	7.829 (1.295)	7.284 (1.650)
60	1.552 (0.342)	3.478 (0.936)	4.024 (1.370)
120	0.936 (0.240)	1.939 (0.376)	2.455 (0.663)
240	0.470 (0.124)	0.956 (0.156)	1.208 (0.302)
480	0.076 (0.075)	0.291 (0.096)	0.364 (0.084)
720	0 (0)	0.078 (0.066)	0.108 (0.070)

## Abbreviations

AE: Adverse event
BMI: Body mass index
IV: Intravenous
MPI: Myocardial perfusion imaging
SD: Standard deviation
